# New insights from integrated bioinformatics analysis: the role of circadian rhythm disruption and immune infiltration in obstructive sleep apnea disease

**DOI:** 10.3389/fimmu.2023.1273114

**Published:** 2023-12-15

**Authors:** Xinyue Zhang, Yixuan Wang, Zhou Pan, Ke Hu

**Affiliations:** Department of Respiratory and Critical Care Medicine, Renmin Hospital of Wuhan University, Wuhan, China

**Keywords:** obstructive sleep apnea, circadian rhythm, immune infiltration, machine learning, bioinformatics analysis

## Abstract

**Background:**

Circadian rhythm disruption and immune infiltration are both closely associated with the development of Obstructive sleep apnea (OSA) disease and a variety of cardiovascular and neurological complications, but their interactions with OSA disease are not clear. In this study, we used bioinformatics to investigate the roles of circadian rhythm disruption and immune microenvironments in OSA.

**Methods:**

We analyzed differential genes and their associated functional pathways in the circadian rhythm-associated OSA dataset, then regrouped OSA samples using the differential genes and explored differences in immune cell infiltration between the two different subgroups. Meanwhile, we used two machine learning algorithms to further define circadian rhythm-related signature genes and to explore the relationship between key genes and immune cell infiltration. Finally, we searched for the transcription factors of the key differential gene JUN.

**Results:**

We screened 15 circadian rhythm-related differential genes in the OSA-related dataset and further defined 3 signature genes by machine learning algorithms. Immunoassays showed a significant increase in resting mast cell infiltration and a decrease in monocyte infiltration in the OSA group. The results of our animal experiments also confirmed that the expression of these 3 key genes, as well as the immune cell infiltration, showed a trend consistent with the results of the bioinformatics analysis.

**Conclusions:**

In conclusion, this study reveals the interaction between circadian rhythm disruption and immune infiltration in OSA, providing new insights into the potential pathogenesis of OSA.

## 1 Introduction

Obstructive sleep apnea (OSA) is a common sleep disorder characterized by recurrent upper airway obstruction during sleep, which leads to the development of intermittent hypoxemia and hypercapnia, ultimately resulting in systemic inflammation, sympathetic activation, and endothelial dysfunction (1). OSA is known to be an independent risk factor for the development of several diseases, including cardiovascular disease, metabolic disorders, and neurocognitive dysfunction (2). The basic pathophysiologic mechanisms underlying the development of OSA are not clearly understood, which creates a very significant impediment to the development of new therapies for OSA disease.

OSA is known to disrupt normal sleep patterns and negatively affect people’s physiological processes (3). Therefore, more and more researchers are interested in the relationship between OSA and circadian rhythms. Circadian rhythms are 24-hour biological clock systems within the human body that regulate a range of physiological processes, including the sleep-wake cycle, hormone secretion, appetite, and metabolism (4). The interactions between OSA and circadian rhythms are complex and most likely bi-directional, with each contributing to the pathophysiology of the other (5). Studies have shown that symptoms associated with circadian rhythm disruption such as fatigue, lethargy, and poor concentration are very common in patients with OSA (6). In addition, common risk factors such as obesity may increase the occurrence of both OSA and circadian rhythm disorders (7). However, the exact relationship between OSA and circadian rhythm disruption remains poorly understood, and future studies could further explore the effects of OSA on circadian rhythms and how to optimize sleep-wake rhythm regulation in patients with OSA.

Circadian rhythms are a fundamental biological process that regulates a variety of physiological functions, including the immune response (8, 9). Many components of the immune system exhibit circadian rhythms, including the expression of multiple cytokines, lymphocyte populations, and phagocytic activity (10–12). The molecular mechanisms underlying the interactions between circadian rhythms and the immune system involve clock genes and clock-control genes, which regulate immune cell function and cytokine release (13). Therefore, understanding the relationship between circadian rhythms and the immune system is important for developing new therapies for OSA diseases.

In this study, we analyzed the differential genes and their associated functional pathways in the circadian rhythm-associated OSA dataset, and then regrouped the OSA samples using the differential genes, and explored the differences in immune cell infiltration between the two different subgroups. Meanwhile, we used two machine learning algorithms to further define circadian rhythm-related signature genes. Next, given that IH is one of the most important pathophysiology of OSA disease as well as most of the basic studies of OSA disease currently use the IH model (14, 15), we chose to use IH rats to simulate OSA disease. qPCR experiments verified the expression of key genes in OSA, which were all of good value for the diagnosis of OSA and closely associated with immune cell infiltration in OSA. Finally, we searched for the transcription factors of the key differential gene JUN.

## 2 Methods

### 2.1 Data download

We downloaded the OSA-related microarray datasets GSE38792 and GSE135917 from the GEO database (http://www.ncbi.nlm.nih.gov/GEO/). where GSE38792 included 10 OSA samples and 8 normal control group samples with sample tissue originating from subjects’ visceral adipose tissue; GSE135917 consists of 34 OSA samples and 8 normal control samples with sample tissue derived from the subjects’ subcutaneous adipose tissue. We de-batched the datasets using the “sva” package and merged datasets GSE38792 and GSE135917. In addition, 2091 circadian rhythm-related genes were obtained from CircaDB (https://circadb.org) and MSigDB (https://www.gsea-msigdb.org/gsea/msigdb) (**Supplementary Table 1**).

### 2.2 Differentially expressed genes (DEGs) analysis

We extracted the expression of 2091 circadian rhythm-related genes in the integrated datasets GSE38792 and GSE135917, and analyzed the differential expression of these genes in OSA and normal control samples using the R package “limma” with the parameters |IogFC|>0. 585, P<0. 05 and plotting heatmaps and volcano maps of DEGs (R packages “ pheatmap”, “ggplot2”).

### 2.3 Functional enrichment analysis

To further investigate the functions of these DEGs, we performed Gene Ontology (GO) enrichment analysis and Kyoto Encyclopedia of Genes and Genomes (KEGG) enrichment analysis on DEGs. We used the R packages “clusterProfiler”, “ggplot2” and “org. Hs. eg. db” to plot the related histograms. Meanwhile, we used the “c2. cp. kegg. v11. 0. symbols” gene set in the molecular signature dataset (MSigDB, https://software.broadinstitute.org/gsea/msigdb) as a reference set to perform GSEA analysis on the OSA group and the normal group in order to explore the two groups between the different biological significance and function.

### 2.4 Consensus clustering analysis

Consensus cluster analysis of circadian rhythm-related DEGs was performed using the R package “ConsensusClusterPlus”, and the samples were categorized into 2 different clusters. Principal component analysis was performed on the two clusters, and the circadian rhythm-related gene scores of the two clusters were compared. In addition, we further analyzed the expression of DEGs in different clusters. Finally, we compared the differences in immune cells between the 2 subclusters.

### 2.5 Machine learning algorithms

To screen for disease-critical genes associated with circadian rhythms, we used DEGs to screen for critical genes associated with circadian rhythms in OSA using a support vector machine (SVM) operator and random forest algorithm (RF). The SVM-RFE algorithm screens for important genes using the “e1071” and “ svmRadial” packages to screen for important genes. Finding the point with the smallest error in the random forest model and then constructing the model to get the importance of the genes, based on the score we chose the top three ranked genes that were greater than 2.5 for subsequent analysis. Finally, the important genes calculated by the two algorithms were intersected to obtain 3 key genes, and we further analyzed the efficay of the 3 key genes in diagnosing OSA and calculated the AUC of the diagnostic ROC for each gene using the “pROC” package to determine the accuracy of the assessment. We screened the key genes by two machine learning methods, and the key gene expression prediction OSA is the correlation probability obtained by calculating the sum of the expression of these genes and the corresponding correlation scores.

### 2.6 Column line diagram of key gene diagnostics

To further apply to clinical work, we use the R package “rmda” to plot nomograms, calibration curves, decision curves, and clinical impact curves for the scoring of characterized genes.

### 2.7 Immune cell infiltration

The relative proportions of 22 types of immune cells were calculated using the CIBERSORT (https://cibersortx.stanford.edu) algorithm, and the immunological scores of each sample were counted using the “ESTIMATE” algorithm. In addition, we further explored the correlation between key genes and immune cell infiltration and demonstrated the correlation by drawing lollipop plots using the “Spearman” and “ggplot2” packages in R software.

### 2.8 Transcription factors (TFs) prediction

TFs are proteins that can bind to specific DNA sequences and regulate gene expression. TRRUST (https://www.grnpedia.org/trrust/) is a manually annotated database of transcriptional regulatory networks. TRRUST contains not only the target genes corresponding to the transcription factors but also the regulatory relationships among the transcription factors. We used this database to obtain key gene-related regulatory relationships and performed the construction of TF-gene regulatory networks by Cytoscape software. Subsequently, we verified the expression levels of these TFs in the integrated datasets GSE38792 and GSE135917 using a t-test.

### 2.9 Animal models

Six- to eight-week-old male SD rats from the Animal Experiment Center of Wuhan University were subjected to a 12-h light/12-h dark cycle for 2 weeks before the start of the experiment, and then were randomly divided into the Sham group or the IH group. In the IH group, rats were exposed to a designed chamber in which the flow rates of nitrogen and oxygen were controlled by a timed solenoid control system. During each 80-s period of the IH cycle, nitrogen was dispensed into the cage for 40 s at a flow rate that reduced the fraction of inspired oxygen (FiO2) to <10% for the first 10 s and then averaged 6-8% FiO2 throughout hypoxia. air was then introduced at a rate that allowed for 21% FiO2 over a 40-s period. These rats were placed in cages for 8 hours per day for 2 weeks. For control exposures, the inflow gas was always room air.

### 2.10 RNA extraction and qRT-PCR

Total RNA from lung tissues was extracted using Trizol according to the manufacturer’s instructions and reverse-transcribed into cDNA using a reverse-transcription kit. qRT-PCR was performed using 2× SYBR Green qPCR ProMix in a real-time PCR detection system (Bio-Rad, USA). The reaction mixture in a 96-well plate was incubated at 95°C for 3 min, followed by 40 cycles at 95°C for 5 s and at 60°C for 30 s. The primer sequences are as follows:

CSRNP1 F:5’-CGAAGGATTGACCGAGAGGAGAAG-3’, R:5’-AGACGCCATCACAGTGACAACC-3’;

JUN F:5’-CGCACCTCCGAGCCAAGAAC-3’, R:5’-GGGTCGGTGTAGTGGTGATGTG-3’;

FAM185A F:5’-AGGTGATGTGGTCTGTCTTGGAAC-3’, R:5’-TGAAGCAAACCATCCTCCGTAGAG-3’.

### 2.11 Immunofluorescence

Fresh lung tissues were paraffin-embedded, and then the tissue sections were incubated with rabbit anti-CD14 antibody (Proteintech, Wuhan, China) and rabbit anti-c-kit antibody (ABclonal, Wuhan, China) primary antibodies at 4°C overnight. Then, they were mixed with secondary antibodies and incubated at room temperature for 50 min away from light. Finally, the nuclei were restained with DAPI and the sections were imaged under a C2t Nikon fluorescence microscope.

### 2.12 Statistical analysis

Data are expressed as mean ± standard deviation (SD) of three independent experiments and were analyzed using GraphPad Prism 9. 0 (GraphPad Inc, San Diego, USA). Student’s t-test was used to assess the differences between the two groups, and a P value <0. 05 was considered statistically significant.

## 3 Results

### 3.1 DEGs analysis

**Figure 1:**
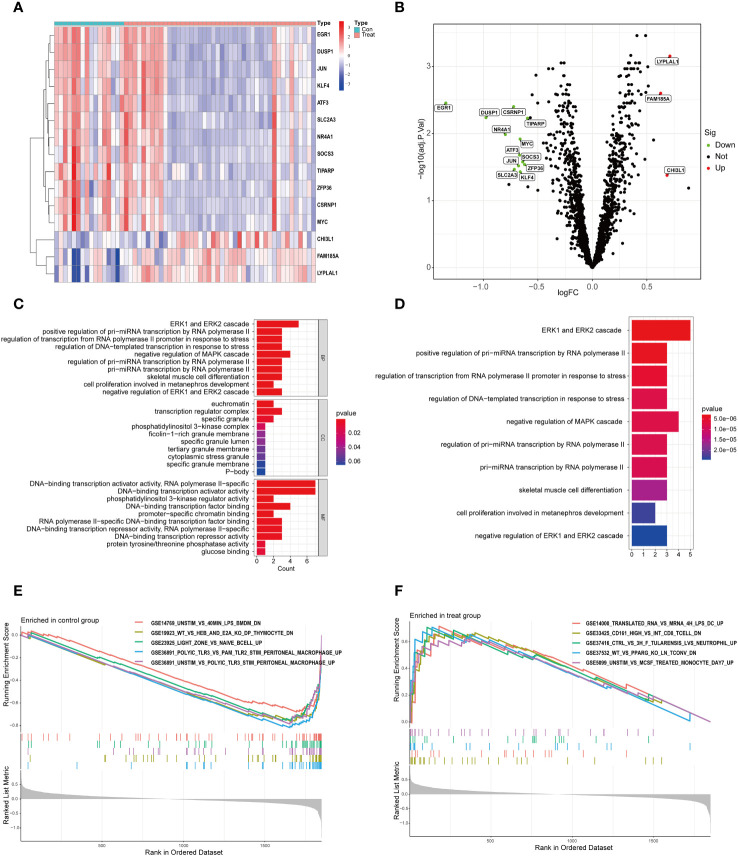
DEGs and functional enrichment analysis in OSA. **(A)** Heat map of DEGs; **(B)** Volcano map of DEGs; **(C)** GO enrichment analysis; **(D)** KEGG enrichment analysis; **(E)** GSEA enrichment analysis in the normal group; **(F)** GSEA enrichment analysis in the OSA group.

We analyzed the differential expression of circadian rhythm-related genes between the OSA and normal control groups and showed that there were 15 DEGs between the 44 OSA and 16 normal groups, of which 3 were up-regulated in the OSA group and 12 were down-regulated in the OSA group (**Figures 1A, B**, **Table 1**).

**Table 1:** Circadian rhythm related DEGs.

id	logFC	AveExpr	t	P. Value	adj. P. Val	B
LYPLAL1	0. 7117156	6. 8861779	5. 3064035	1. 52E-06	0. 0007006	5. 0658382
FAM185A	0. 6266798	6. 6869997	4. 3212496	5. 57E-05	0. 0025117	1. 7291864
EGR1	-1. 344502	9. 3242776	-4. 087412	0. 0001249	0. 0035129	0. 9868371
CSRNP1	-0. 72217	7. 5669655	-4. 031463	0. 000151	0. 0039798	0. 8126272
DUSP1	-0. 976154	10. 652258	-3. 824457	0. 0003015	0. 0058066	0. 180293
TIPARP	-0. 595324	8. 2546407	-3. 80905	0. 0003172	0. 0059234	0. 1340274
NR4A1	-0. 798303	8. 2053555	-3. 541582	0. 0007514	0. 0103676	-0. 650377
MYC	-0. 662862	8. 2044819	-3. 451938	0. 0009954	0. 012109	-0. 904973
ATF3	-0. 668596	7. 5246353	-3. 148554	0. 0025005	0. 021001	-1. 733278
SOCS3	-0. 639059	7. 7347085	-3. 011064	0. 0037334	0. 0265505	-2. 090713
ZFP36	-0. 622328	7. 8203087	-2. 962491	0. 0042903	0. 0290336	-2. 21418
JUN	-0. 677171	9. 2700333	-2. 940435	0. 0045678	0. 0304206	-2. 269747
SLC2A3	-0. 715899	8. 9501888	-2. 867677	0. 0056057	0. 0343208	-2. 450837
KLF4	-0. 66178	8. 6705399	-2. 827415	0. 0062696	0. 037275	-2. 549561
CHI3L1	0. 6853124	8. 1096147	2. 7531477	0. 0076878	0. 0420556	-2. 728851

### 3.2 Functional enrichment analysis

To further investigate the functions of these DEGs, we performed an enrichment analysis. The most enriched GOs were categorized into biological processes (BP), cellular components (CC), and molecular functions (MF), which mainly included cell growth, proliferation, differentiation, apoptosis, stress response, and activation of genes involved in gene transcription (**Figure 1C**). Apoptosis and stress response lead to infiltration of immune cells in the body (16). And cell growth, proliferation and apoptosis all exhibit circadian rhythms (17). Also, the progression of OSA disease involves apoptosis and stress response (18, 19); KEGG analysis showed that these DEGs were mainly enriched in cell growth, proliferation, cellular stress response, and adaptive response as well as miRNA biosynthesis and gene regulation (**Figure 1D**). Finally, we compared the GSEA pathway enrichment in the gene sets of the OSA and normal groups, and showed that the OSA group was mainly involved in genes related to inflammatory response, immune cell subpopulations, tularemia infections, PPARγ, and monocytes; whereas the normal group was mainly involved in genes related to LPS-stimulated macrophages, DP-type thymocytes, superficial zones in the lymph nodes with inactivated B cells, and PolyIC-stimulated of abdominal macrophages and other related genes (**Figures 1E, F**).

### 3.3 Consensus cluster analysis

**Figure 2:**
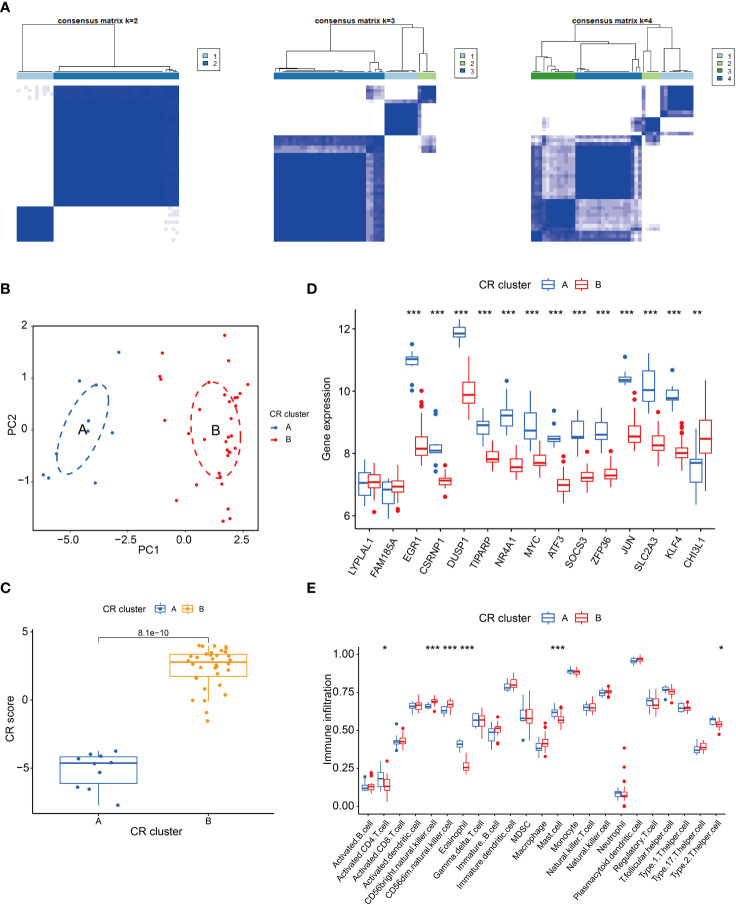
Cluster analysis of circadian rhythm-related genes. **(A)** Heatmap of cluster analysis; **(B)** PCA analysis; **(C)** Box line plots of circadian rhythm gene scores in the 2 subpopulations; **(D)** Expression of DEGs in the 2 subpopulations; **(E)** Differences in immune cells in the 2 subpopulations. *P < 0.05, **P < 0.01, ***P < 0.001.

**Figure 3:**
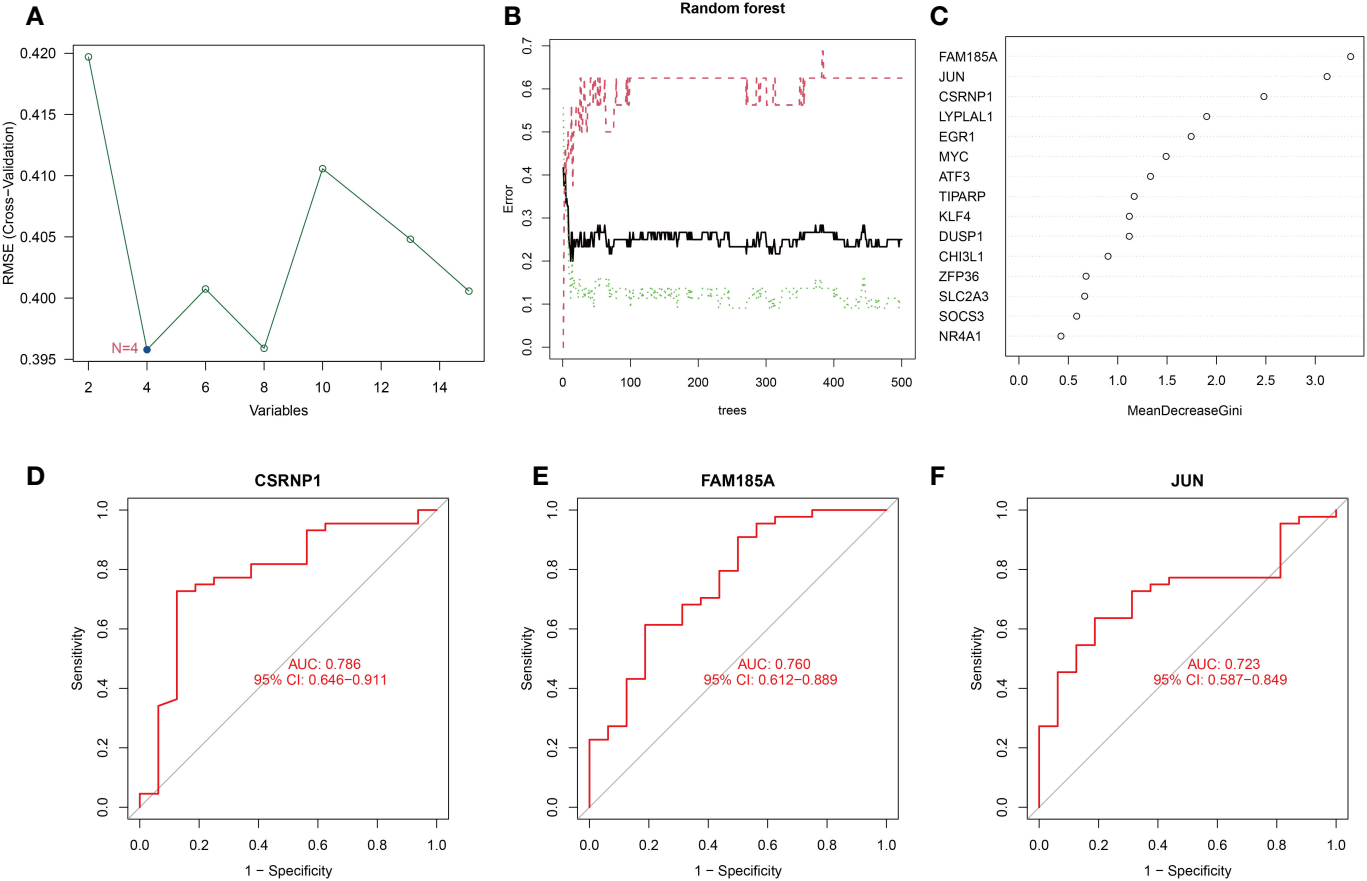
Screening and evaluation of key genes. **(A)** Filtering of 15 DEGs with SVM-RFE algorithm to identify key genes; **(B, C)** Filtering of 15 DEGs with RF algorithm to identify key genes; **(D–F)** ROC curves of key gene diagnosis.

Clustering of DEGs based on their expression differences between groups allows for more isotropically expressed genes inside the clusters to be in the same cluster. As shown in the **Figure 2A**, 3 or more clusters were not good enough to distinguish subgroups, so we chose to cluster the OSA samples into 2 subgroups using 15 DEGs associated with circadian rhythms, and to test the clustering effect, we utilized principal component analysis and found that the 2 subgroups could be well distinguished (**Figure 2B**). Meanwhile, it is worth noting that we constructed a scoring system using circadian rhythm genes, and the scores between subgroups A and B were similarly different (**Figure 2C**). To further understand the expression of these 15 circadian rhythm-related genes between the two subpopulations, we performed a difference analysis, which showed that LYPLAL1 and FAM185A did not differ between the 2 subpopulations, whereas all of the genes were down-regulated in cluster B except for CHI3L1, which was up-regulated in cluster B (**Figure 2D**). Finally, we analyzed the expression of immune cells between the 2 subpopulations. CD56bright. natural. killer. cell and CD56dim. natural. killer. cell had increased infiltration in cluster B, whereas Activated. CD4. T. cell, Mast. cell, Eosinophil and Type. 2. T. helper. cell were infiltrated less in cluster B (**Figure 2E**).

### 3.4 Screening of key genes

To further identify the key genes of OSA associated with circadian rhythms, we screened the DEGs using two algorithms, SVM-RFE and RF. Although the RMSE values of N=4 and N=8 were somewhat close to each other as can be seen in **Figure 3A**, the RMSE value of N=4 is actually still lower than that of N=8. Therefore, we finally chose the RMSE value of N=4 for our study, and the four characterized genes obtained were LYPLAL1, CSRNP1, JUN, and FAM185A; We use the permutation importance (mean decrease accuracy) to calculate the importance of features. meanwhile, the RF method screened for the genes with significance greater than 2. 5, and finally CSRNP1, JUN and FAM185A were obtained (**Figures 3B, C**). We integrated the results of the two algorithms and finally took the intersection to obtain the 3 key circadian genes in OSA (CSRNP1, JUN, and FAM185A). Meanwhile, to better test the efficacy of these 3 key genes in diagnosing OSA, we plotted the ROC curves of the diagnosis of these 3 genes, and the results showed that the AUC of the 3 genes in diagnosing OSA was greater than 0. 70, which indicated that all of them were of good value for the diagnosis of OSA (**Figures 3D–F**).

**Figure 4:**
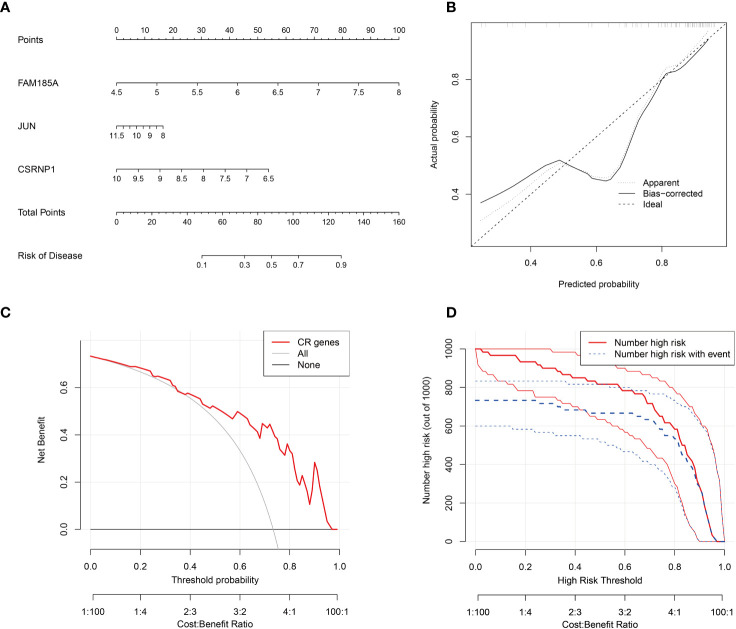
Key genes nomogram model. **(A)** nomogram of characterized genes; **(B)** calibration curves of the model; **(C)** clinical decision curves; **(D)** clinical impact curves (the red curve (Number high risk) indicates the number of people classified as positive (high risk) by the model at each threshold probability; the blue curve (Number high risk with the event) is the number of true positives at each threshold probability).

To get a more clinically applicable diagnostic model, we constructed a nomogram of these 3 characterized genes (**Figure 4A**), and based on the scoring of each gene expression, we can get the risk percentage of the patient, and of course, we calibrated the reliability of the nomogram, and the results were good. The calibration curve for the clinical model shows a good fit for the model and the decision curve shows a higher degree of benefit from the model in diagnostic situations and a higher probability of diagnosing a positive patient (**Figures 4B–D**).

### 3.5 Immune cell infiltration analysis

**Figure 5:**
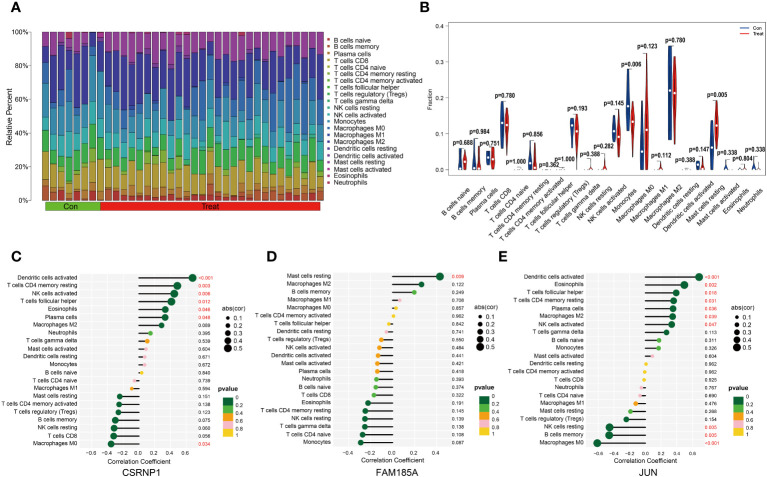
Immune cell infiltration analysis. **(A, B)** The difference in immune cell infiltration between OSA and normal groups; **(C–E)** Lollipop plot of correlation between key genes and immune cell infiltration.

Immune cell infiltration plays a pivotal role in various diseases. In our study, we delved into the disparities in immune cell infiltration between the OSA group and the normal control group. Our findings illustrated that Monocytes demonstrated a pronounced infiltration in the control group, whereas the proportion of Mast cells resting in the infiltration was significantly higher in the OSA group, and the rest of the immune cells were not found to be different between the two groups (**Figures 5A, B**). We further explored the relationship between the expression of key genes and immune cell infiltration. We observed that CSRNP1 expression exhibited a positive association with Plasma cells, Eosinophils, T cells follicular helper, NK cells activated, T cells CD4 memory resting and Dendritic cells activated, but it shared a negative relation with Macrophages M0 (**Figure 5C**); The expression of FAM185A was found to be directly related to the infiltration of Mast cells resting (**Figure 5D**); JUN showed a positive linkage with several immune cells, including NK cells activated, Macrophages M2, Plasma cells, T cells CD4 memory resting, T cells follicular helper, Eosinophils, and Dendritic cells activated. However, an inverse relationship was observed with Macrophages M0, B cells memory, and NK cells resting (**Figure 5E**).

### 3.6 TFs prediction

**Figure 6:**
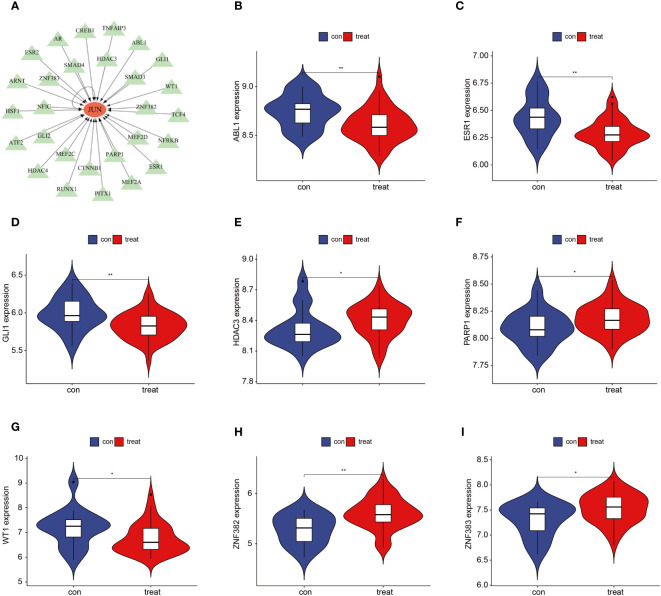
JUN-associated TFs. **(A)** Regulatory network of JUN-associated TFs; **(B–I)** JUN-associated TFs that differed between OSA and controls. *P < 0.05, **P < 0.01.

We further investigated the TFs associated with the 3 key genes and found that no corresponding TFs were found for FAM185A and CSRNP1. There were a total of 28 TFs associated with JUN, including ABL1, AR, ARNT, ATF2, CREB1, CTNNB1, ESR1, ESR2, GLI1, GLI2, HDAC3, HDAC4, HSF1, MEF2A, MEF2C, MEF2D, NFIC NFRKB, PARP1, PITX1, RUNX1, SMAD3, SMAD4, TCF4, TNFAIP3, WT1, ZNF382, and ZNF383 (**Figure 6A**). We compared the differences in the expression of these TFs between the OSA and control groups and showed that ABL1, ESR1, GLI1, and WT1 were down-regulated in the OSA group, whereas HDAC3, PARP1, ZNF382, and ZNF383 were up-regulated in the OSA group (**Figures 6B–I**).

### 3.7 Expression of characterized genes and immune cell infiltration in animal experiments

**Figure 7:**
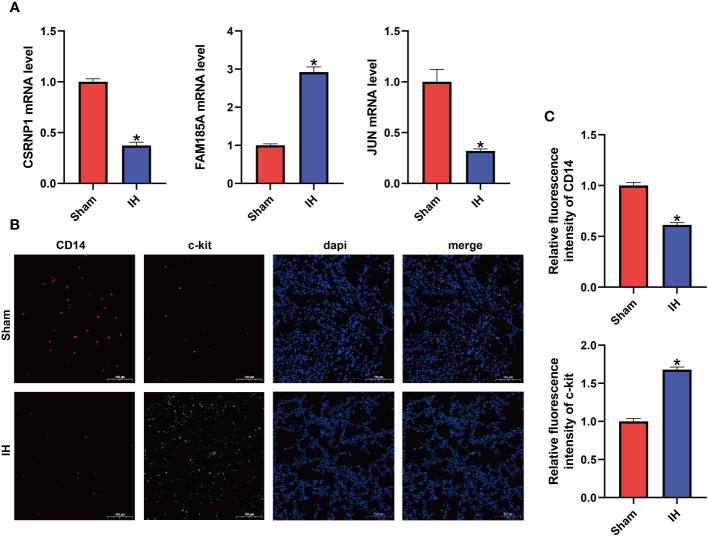
Characteristic gene expression and immune cell infiltration. **(A)** mRNA expression of CSRNP1, JUN, and FAM185A in the lungs of two groups of rats; **(B)** Double immunofluorescence showing CD14 (red light) and c-kit (green light) expression; **(C)** Relative fluorescence intensity of CD14 and c-kit. *P < 0.05.

The mRNA expression of the three characterized genes (CSRNP1, JUN, and FAM185A) was verified by qPCR in rat lung tissues. As shown in **Figure 7A**, the mRNA expression of both CSRNP1 and JUN was significantly lower (P < 0. 05) in the IH group compared with the Sham group, whereas the mRNA expression of FAM185A was significantly higher (P < 0. 05) in the IH group. Next, we verified the immune cell infiltration between the Sham and IH groups by double immunofluorescence staining (**Figures 7B, C**), which showed that the Monocytes marker CD14 was significantly infiltrated in the Sham group (P < 0. 05), whereas c-kit, which marks the Mast cells resting, was infiltrated in a significantly higher proportion in the IH group (P < 0. 05).

## 4 Discussion

OSA is a sleep disorder characterized by the occurrence of upper airway obstruction and apnea during sleep, and it may share risk factors with circadian rhythm disorders and immune-related diseases, such as obesity and metabolic syndrome (20, 21). However, considering that the exact roles of circadian rhythm disruption and immune cell infiltration in OSA disease and their underlying mechanisms are still unclear, we utilized a combined analysis of machine learning algorithms and bioinformatics to obtain 15 DEGs associated with circadian rhythms from an OSA-associated microarray dataset. Based on this study, our study was aimed at analyzing the role of circadian rhythm disruption and immune dysregulation in OSA disease development.

IH is a hallmark manifestation of OSA, and IH is closely related to circadian rhythm disruption. Adamovich et al. studied the rhythmic changes of oxygen and carbon dioxide in mice and found that the oxygen and carbon dioxide levels in mice changed during the dark phase, and at the same time, the expression of clock genes in their body was also altered (22). Some studies have also confirmed that hypoxia interferes with the expression of circadian genes (23). Considering the interconnectedness of OSA and circadian rhythms, we performed GO, KEGG, and GSEA enrichment analyses on these 15 differential genes, and the results showed that these genes were mainly associated with immunity, inflammation, and cell growth. To further screen the disease signature genes, we used two machine learning algorithms, SVM-RFE and RF, to finally obtain three signature genes, CSRNP1, JUN, and FAM185A. Because of the limited datasets related to OSA, we did not find other suitable datasets for validation, so we further validated this result by animal experiments. Next, we verified the expression of these three signature genes by qPCR, and the results showed that the expression trend was consistent with the previous bioinformatics analysis. These three circadian rhythm-related genes have good diagnostic value in OSA disease, and how they specifically regulate the development of OSA disease deserves further investigation. CSRNP1 is a nuclear protein involved in various biological processes such as transcriptional regulation, DNA repair, and cell proliferation, while its expression was down-regulated in microarray studies of various tumors, suggesting that this gene is a potential tumor suppressor gene (24, 25). JUN is a type of TFs involved in the regulation of various biological processes such as cell proliferation, differentiation, and inflammatory responses, and studies have shown that JUN is involved in the multiple immune cell functions and regulation of immune responses (26). We also compared the differences in the expression of JUN-related TFs between the OSA and control groups and showed that ABL1, ESR1, GLI1, and WT1 were downregulated in the OSA group, whereas HDAC3, PARP1, ZNF382, and ZNF383 were upregulated in the OSA group. To date, there are limited studies on the FAM185A gene, which has been suggested to be associated with plexus-forming angiogenesis in fetal lung tissue (27). There is still uncertainty about the exact mechanism of these three characterized genes in OSA disease, and we hope that this study will provide new targets for future basic experimental studies to develop new therapies for OSA.

Based on these 15 circadian rhythm-related DEGs, we conducted an unsupervised consistency clustering study on OSA patients. 44 OSA patients were classified into two subtypes, and principal component analysis revealed that the 2 clusters could be well differentiated. Meanwhile, it is worth noting that we constructed a scoring system using circadian genes, and scores between clusters A and B were also different. To further understand the expression of the 15 DEGs between the two clusters, we performed a difference analysis, which showed that LYPLAL1 and FAM185A did not differ between the 2 groups and all other DEGs were significantly different. In addition, these two subtypes showed different immune cell infiltration in the immune microenvironment. The majority of immune cells express circadian genes and show 24 h rhythmic changes. Numerous studies have emphasized the important link between circadian regulation and the immune system, such as lymphocyte development, leukocyte counts, and cytokine secretion (8). Due to the rhythmic oscillatory changes of endogenous biological clock genes in immune cells, the type of immune response produced by the body is closely related to the time state in which it is located (28). Thus, circadian regulation plays a crucial role in physiology, and it may serve as a key regulator of specific immune functions.

There is a close relationship between immune cell infiltration and OSA disease. OSA may cause enhanced inflammatory response as well as immune cell activation, which adversely affects the body’s immune system, thus contributing to the development of multi-systemic diseases (1, 29). We explored the differences in immune cell infiltration between the OSA group and the normal control group and showed that Monocytes were significantly infiltrated in the control group, while Mast cells resting had a significantly higher percentage of infiltration in the OSA group. A 2018 study found that intermittent hypoxia increased monocyte adhesion and chemotaxis (30). In addition, monocytes may be involved in the increased oxidative stress response and the release of inflammatory cytokines and adhesion molecules in patients with OSA (31). Few studies have reported the relationship between resting mast cells and OSA, and the activation of resting mast cells promotes an increased inflammatory response, which may be involved in the pathophysiologic processes associated with OSA (32).

In conclusion, both circadian rhythm disruption and immune cell infiltration can severely affect OSA disease progression, and their relationship remains an active area of research. Meanwhile, IH is an important factor contributing to the development of OSA disease, which can also further aggravate circadian rhythm disruption and immune cell infiltration. The use of IH model is the most common way to study OSA disease (33). Since most immune cells exhibit circadian rhythms, whether regulating circadian rhythm-related genes could further manage disease progression in OSA by improving the immune response deserves further investigation.

## Data availability statement

The original contributions presented in the study are included in the article/**Supplementary Material**. Further inquiries can be directed to the corresponding author.

## Ethics statement

The animal studies were approved by the Institutional Animal Care and Use Ethics Committee of Renmin Hospital of Wuhan University. The studies were conducted in accordance with the local legislation and institutional requirements. Written informed consent was obtained from the owners for the participation of their animals in this study.

## Author contributions

XZ: Conceptualization, Investigation, Writing – original draft. YW: Methodology, Visualization, Writing – original draft. ZP: Data curation, Resources, Writing – original draft. KH: Funding acquisition, Supervision, Writing – review & editing.
