# Supplementary material for: New insights from integrated bioinformatics analysis: the role of circadian rhythm disruption and immune infiltration in obstructive sleep apnea disease
Source: Front Immunol. 2023 Dec 15;14:1273114. doi: 10.3389/fimmu.2023.1273114 (PMC10758485; doi:10.3389/fimmu.2023.1273114)
Supplement: Supplementary file 1 [file Presentation_1.pdf]

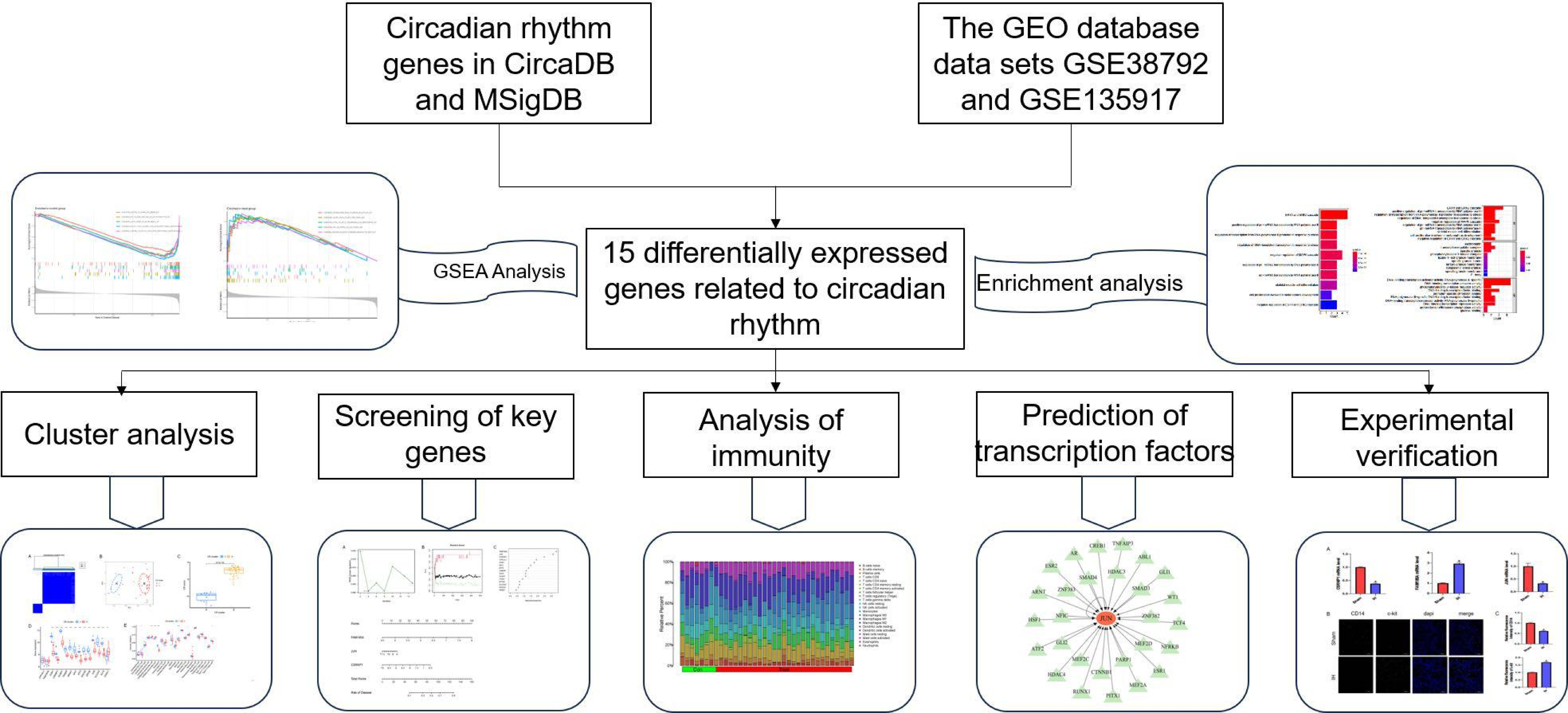
Circadian rhythm genes in CircaDB and MSigDB
The GEO database data sets GSE38792 and GSE135917
GSEA Analysis
15 differentially expressed genes related to circadian rhythm
Enrichment analysis
Cluster analysis
Screening of key genes
Analysis of immunity
Prediction of transcription factors
Experimental verification
